# Quantitative analysis of endobronchial ultrasound elastography in computed tomography‐negative mediastinal and hilar lymph nodes

**DOI:** 10.1111/1759-7714.13579

**Published:** 2020-07-21

**Authors:** Keigo Uchimura, Kei Yamasaki, Shinji Sasada, Sachika Hara, Issei Ikushima, Yosuke Chiba, Takashi Tachiwada, Toshinori Kawanami, Kazuhiro Yatera

**Affiliations:** ^1^ Department of Respiratory Medicine University of Occupational and Environmental Health Fukuoka Japan; ^2^ Department of Respiratory Medicine Tokyo Saiseikai Central Hospital Tokyo Japan

**Keywords:** Bronchoscopy, elastography, endobronchial ultrasound‐guided transbronchial needle aspiration (EBUS‐TBNA), lung cancer

## Abstract

**Background:**

Endobronchial ultrasound (EBUS) elastography assists in the differentiation of benign and malignant lymph nodes (LNs) during transbronchial needle aspiration (TBNA). However, previous studies have not compared B‐mode sonographic images (BSIs) and EBUS elastography images (EEIs) with final pathological diagnoses in radiologically normal‐sized (computed tomography [CT]‐negative) LNs.

**Methods:**

Consecutive patients with CT‐negative LNs, who received EBUS‐TBNA, were retrospectively reviewed. Images of BSIs and EEIs of each LN were stored and independently evaluated. EEIs were assessed by calculating the stiffness area ratio (SAR, blue/overall areas). The receiver operating characteristic curve was used to calculate the cutoff value for the SAR. Diagnostic test parameters were evaluated for each EBUS finding.

**Results:**

A total of 132 patients (149 LNs) were enrolled, and the median SAR of malignant LNs was significantly higher than that of benign LNs (0.58 vs. 0.32, *P* < 0.001). At the SAR cutoff of 0.41, the sensitivity, specificity, positive predictive value, negative predictive value (NPV), and diagnostic accuracy rate (DAR) of elastography were 88.2%, 80.2%, 78.9%, 89.0%, and 83.9%, respectively. The logistic regression analysis showed that elastography was the strongest predictor of malignancy (odds ratio, 18.5; 95% confidence interval [CI]: 6.48–52.6; *P* < 0.001). The highest NPV (96.6%) was achieved with a combination of BSIs and EEIs.

**Conclusions:**

EBUS elastography predicted malignant LNs with a high DAR and NPV in CT‐negative LNs. The NPV was highest when EEIs were combined with BSIs. Therefore, the combined evaluation of CT‐negative LNs using EEIs and BSIs may help bronchoscopists perform EBUS‐TBNA more efficiently.

**Key points:**

**Significant findings of the study:**

Endobronchial ultrasound elastography accurately predicted malignancy with a high diagnostic accuracy rate and negative predictive value in radiologically normal‐sized lymph nodes. The additional use of B‐mode sonographic features resulted in a higher negative predictive value.

**What this study adds:**

Endobronchial ultrasound elastography can guide the accurate collection of specimens with transbronchial needle aspiration, even in radiologically normal‐sized lymph nodes. It can also readily distinguish benign and malignant lymph nodes, thus avoiding unnecessary punctures.

## Graphical abstract

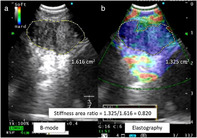

## Introduction

Endobronchial ultrasound‐guided transbronchial needle aspiration (EBUS‐TBNA) has developed into the most widely used standard sampling technique for mediastinal and hilar lesions, irrespective of their benign or malignant status.^1^, ^2^, ^3^ Major international guidelines recommend that EBUS‐TBNA should be performed before mediastinoscopy, especially for the pathological diagnosis and staging of lymph nodes (LNs) in lung cancer.^4^, ^5^, ^6^

Recently, one cohort study reported that proper preoperative staging was associated with survival benefits in patients with non‐small cell lung cancer (NSCLC).^7^ The superiority of EBUS‐TBNA over radiological imaging, such as computed tomography (CT) and positron emission tomography‐CT (PET‐CT), has been attributed to its higher diagnostic accuracy rate (DAR).^4^ Two meta‐analyses reported a cumulative sensitivity and specificity of 88%–93% and 100%, respectively, for EBUS‐TBNA in NSCLC staging; however, targeted LNs had a short axis diameter of at least 1 cm on CT in the majority of the included studies.^8^, ^9^ Two additional meta‐analyses which evaluated the use of EBUS‐TBNA for radiologically normal‐size (CT‐negative) LNs in NSCLC patients, reported high negative predictive values (NPVs), ranging from 91% to 93%.^10^, ^11^ Therefore, the utility of EBUS‐TBNA for CT‐negative LNs has already been established.

For diagnosing and staging with EBUS‐TBNA in patients with lung cancer, it is necessary to accurately select the LNs to be punctured. B‐mode sonographic findings (size, shape, echogenicity, margin, central hilar structure [CHS], and coagulation necrosis) and vascular patterns on power Doppler mode have previously been reported as useful EBUS parameters for distinguishing benign and malignant LNs.^12^, ^13^

Elastography is an ultrasonographic method for generating objective strain images of tissue compressibility. EBUS elastography has made it possible to detect and colorize the hardness of LNs. Hard tissues are shown in blue, while intermediate tissues are shown in green, and soft tissues are shown in yellow or red. A recent meta‐analysis characterized the differences between benign LNs and malignant LNs when evaluated using EBUS elastography.^14^ Quantitative elastography measures include the stiffness area ratio (SAR, blue area/overall area) or mean strain value of the LN, and the strain ratio between the LN and surrounding tissues. Qualitative elastography measures include the 3‐ and 5‐image pattern classifications.^14^, ^15^, ^16^, ^17^, ^18^, ^19^, ^20^, ^21^, ^22^, ^23^ The 3‐image pattern classification is often problematic, as the definition of the pattern is ambiguous. In general, unlike qualitative tests, quantitative tests can avoid the problems associated with subjective evaluations by using specific numerical values.

Elastography has been previously used in the breast, thyroid, liver, pancreas, and gastrointestinal tract.^24^, ^25^, ^26^, ^27^, ^28^, ^29^ Small lesion size has been reported to cause false results on both breast ultrasonography with elastography.^28^ However, no prior studies of bronchoscopy on CT‐negative LNs have compared B‐mode sonographic images (BSIs) and EBUS elastography images (EEIs) with the final pathological diagnoses.

The aim of this study was to evaluate the diagnostic utility of EBUS elastography, and identify the most accurate EBUS parameters for the differentiation of benign and malignant CT‐negative LNs.

## Methods

### Patients

The medical records of consecutive patients with mediastinal and/or hilar LNs, who underwent EBUS‐TBNA at the university hospital of the University of Occupational and Environmental Health, Japan (UOEH) between April 2015 and November 2018, were retrospectively analyzed. Targeted LNs that underwent EBUS‐TBNA had enlargements on chest CT (short axis diameter ≥ 5 mm) or high [18F]‐fluorodeoxyglucose (FDG) uptake on PET‐CT (maximum standardized uptake value, SUVmax ≥2.5). CT‐negative LNs were defined by a short axis diameter of less than 1 cm on chest CT. Before EBUS‐TBNA, each patient underwent thin section CT, using a 1 mm slice thickness. This study was approved by the institutional review board (No. H30‐074), and written informed consent was obtained from all participants. EBUS‐TBNA was used when clinically required, and was not performed solely for the purposes of this study.

### CT and PET‐CT scan protocol

Chest CT scans were performed using a single source 32‐detector or 64‐detector CT scanner (Aquilion 32 or 64, Toshiba Medical Systems, Otawara, Japan). The settings were as follows: 1 mm collimation, 0.5 second rotation time, 27 or 53 pitch (ratio of table movement per rotation to total beam width), and 120 kV. The noise level was set at 10 standard deviations using automatic tube current modulation (*z*‐axis modulation with Real E.C. technique, Toshiba Medical Solutions). All PET‐CT images were obtained using a PET‐CT scanner (True Point Biograph 16, Siemens Healthcare, Tokyo, Japan). Patients received an intravenous administration of 4.0 MBq/kg 18F‐FDG in the sitting position after fasting for 6 hours, and a PET‐CT scan 60 minutes later. Chest CT images were reviewed by two pulmonologists (KU and SH) who were blinded to the results of the final pathological diagnoses, as well as the BSIs and EEIs. PET‐CT images were evaluated by an independent and blinded radiologist.

### EBUS‐TBNA procedure

All patients underwent bronchoscopy under local anesthesia using sedatives and intravenous midazolam for conscious sedation. EBUS‐TBNA was performed using a convex probe ultrasound bronchoscope (BF‐UC260FW, Olympus, Tokyo, Japan) and a 22‐gauge needle (Vizishot® NA‐201SX‐4022, Olympus, Japan, or Expect™ Pulmonary E00558220, Boston Scientific Corporation, Natick, MA, USA). All procedures were performed by residents under the supervision of a bronchoscopist (KU) with sufficient experience, as previously reported.^4^, ^6^ First, the pharynx was locally anesthetized with 8% lidocaine spray (approximately 0.2 mL). All patients subsequently underwent oral bronchoscopy without intratracheal intubation. Prior to EBUS‐TBNA, vocal cords, trachea, and bronchi were intermittently nebulized with 1% lidocaine via a bronchoscope.

A dedicated ultrasound processor (EU‐ME2 PREMIER, Olympus, Tokyo, Japan) was used to evaluate EBUS images in B‐mode sonography and EBUS elastography, and vascular patterns were evaluated with power Doppler ultrasound. The target LN was evaluated in B‐mode ultrasonography at its maximum diameter, and the images were stored in JPEG format. EEIs evaluated at the same location were also stored in JPEG format, as previously reported.^12^, ^15^, ^16^, ^18^ After confirming the absence of large blood vessels using power Doppler ultrasound at the puncture site, targeted LNs were punctured using stylets. EBUS‐TBNA was performed with adequate negative pressure and 20–30 strokes per puncture. Each approach of a targeted LN involved three EBUS‐TBNA punctures, or two core tissue samples were collected as a standard. Rapid on‐site cytology evaluations were not performed for all patients.

### EBUS image findings and pathological diagnosis

The BSIs and EEIs were reviewed by two pulmonologists (KU and SH), who were blinded to the results of the pathological diagnoses and CT images. Based on a previously reported BSI classification,^12^ the BSIs were evaluated for the following parameters: size (short‐axis diameter > 1 cm or ≤ 1 cm), shape (round or oval), echogenicity (heterogeneous or homogeneous), margins (distinct or indistinct), CHS (presence or absence), and coagulation necrosis sign (CNS) (presence or absence). A round shape was defined as a ratio of the short to long axis diameters of <1.5. A distinct margin was defined as one in which the margin between the LN and the surrounding tissues was clearly visible (i.e., above 50%). A CHS was defined as a flat, linear, hyperechoic region found in the center of the LN. A CNS was defined as a hypoechoic region, without blood flow, within the LN. Each sonographic feature was compared with the final pathological results. As previously reported,^12^, ^18^ B‐mode sonographic features were used to characterize LNs as benign, based on the absence of a (i) round shape; (ii) heterogeneous echogenicity; (iii) distinct margin; and (iv) CNS. If any of these four findings were positive, LNs were judged as suggestive of malignancy.

**Figure 1:**
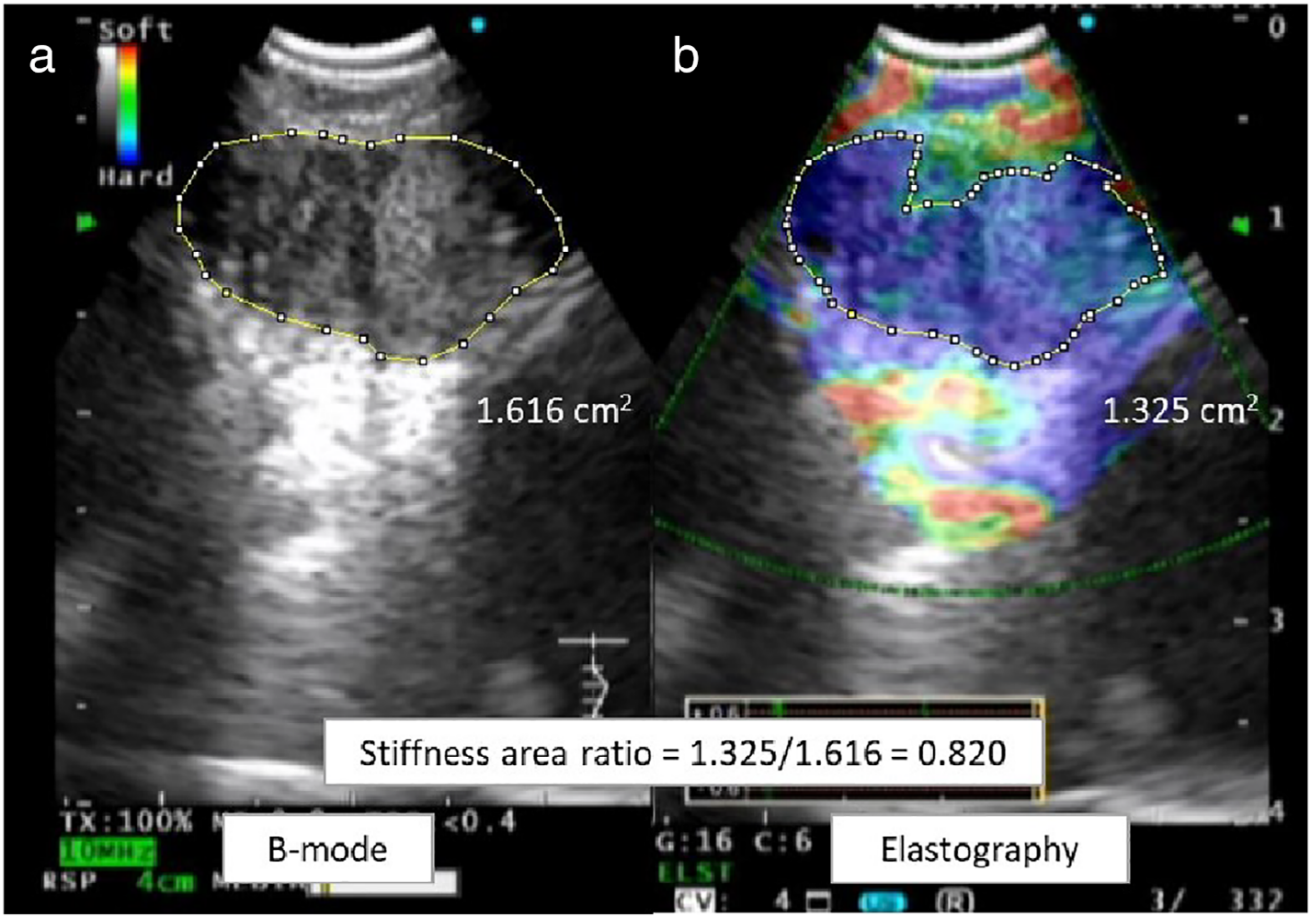
Quantitative evaluation of endobronchial ultrasound (EBUS) elastography using ImageJ. The stiffness area ratio was calculated by dividing the blue area (indicating stiffness) by the entire area of the lymph node. (**a**) The entire area of the target lymph node was manually enclosed and measured on the B‐mode sonographic image. (**b**) The stiffness area within the lymph node was manually enclosed and measured on the EBUS elastography image. The stiffness area ratio was computed after EBUS‐transbronchial needle aspiration.

The image analysis software program ImageJ 1.45s^30^ (National Institutes of Health, Bethesda, MD, USA) was used to evaluate EEIs and determine the SAR of the target LNs, as the previous report (Fig 1).^22^ To measure the area and calculate the SAR, we analyzed the image using ImageJ 1.45s software. (i) We opened the JPEG file containing both the BSIs and EEIs by selecting “Open” in the “File” menu. (ii) To set the scale, we selected the “Straight” tool from the toolbar and manually drew a line on the 1 cm scale of the EBUS image. We then selected “Set Scale” from the “Analyze” menu and set the “Known distance” to “1” and the “Unit of length” to “cm” in the set scale dialog box. (iii) Subsequently, we selected the “Polygon selections” tool from the toolbar and manually drew an outline of the area to be evaluated. We then selected “Measure” from the “Analyze” menu and recorded the area value displayed in “Results;” and (iv) Finally, the SAR was calculated by dividing the blue area (indicating hard tissues) by the entire area of the LN. The final pathological results were compared with the SAR. A cutoff value for the SAR and the area under the curve (AUC) were identified from the receiver operating characteristic (ROC) curve. In three prior cohort studies, the cutoff values of the SAR were used to distinguish benign and malignant LNs on EBUS elastography.^16^, ^18^, ^22^ When the SAR of the target LN was higher than the cutoff value, the LN was judged as malignant on EBUS elastography.

The evaluation of the utility of a combination of elastographic and B‐mode sonographic features was based on a previous retrospective study.^18^ LNs were only judged as suggestive of malignancy (or a benign process) if they were judged as such in both assessments. If the assessments of the elastographic and B‐mode sonographic features were conflicting, the lymph node was excluded from the calculation of the diagnostic test parameters.

Histological and cytological specimens were evaluated by an independent cytologist and pathologist blinded to the BSIs and EEIs. The final diagnosis of each LN was based on the pathological evidence obtained from EBUS‐TBNA, surgery, and bacterial culture, or the demonstration of the absence of disease progression on clinical and radiological follow‐up (at least six months).

### Statistical analysis

Data are presented as frequency, median (range), and percentage. The sensitivity, specificity, positive predictive value (PPV), NPV, and DAR were calculated for each LN according to standard definitions. ROC analysis and Youden index (sensitivity + specificity − 1) were used to calculate the optimal cutoff for the SAR value. Based on a two‐sided hypothesis, comparisons between the two groups were examined using the Fisher's exact test or Mann‐Whitney U test. All B‐mode sonographic and EBUS elastographic parameters, which were potentially predictive of malignant LNs,^12^, ^14^ were analyzed with multivariate logistic regression. The Cochran‐Armitage test was used to identify the trend with one‐sided *P*‐values. *P*‐values <0.05 were considered statistically significant. The statistical analyses were performed with EZR^31^ (Saitama Medical Center, Jichi Medical University, Saitama, Japan), which is a graphical user interface for R (The R Foundation for Statistical Computing, Vienna, Austria, v2.13.0), and a modified version of R commander (v1.8–4).

## Results

### Patients and lymph nodes

Between April 2015 and November 2018, EBUS‐TBNA was performed in 464 patients (663 LNs) at the UOEH hospital. A total of 143 (164 CT‐negative LNs) patients underwent EBUS‐TBNA; among these patients, six (eight LNs) with insufficient follow‐up and five (seven LNs) with missing imaging data were excluded. Thus, 132 patients (149 LNs) were eventually included in the study.

Table 1 summarizes the baseline characteristics of the patients and LNs, as well as the final pathological diagnoses. The patients included 91 men (68.9%), and the overall median age was 71 years. In terms of comorbidities, eight patients (6.1%) had a history of pulmonary tuberculosis, and 40 patients (30.3%) had a history of occupational inhalation exposure. The median LN size on CT was 8.0 (4.0–9.9) mm, and the median LN size on EBUS was 8.0 (4.9–16.0) mm. The LNs included 79 (53%) subcarinal lymph nodes (station 7) and 50 (33.6%) lower paratracheal lymph nodes (station 4R or 4L). The final diagnoses included 68 (45.6%) malignant and 81 (54.4%) benign lesions. PET‐CT was performed on 113 LNs (75.8%); 89 LNs (59.7%) had a SUVmax ≥2.5. None of the patients suffered complications related to the EBUS‐TBNA procedure.

**Table 1:** Baseline characteristics of the radiologically normal‐sized lymph nodes assessed by EBUS‐TBNA

Characteristics	Numbers of lymph nodes (*N* = 149)	(%)
Patients, (n)	132	
Sex		
Male	91	(68.9)
Female	41	(31.1)
Age, years (median, range)	71 (27–87)	
Comorbidity		
History of pulmonary tuberculosis	8	(6.1)
Occupational inhalation exposure history	40	(30.3)
Dust (mineral, metal, coal, and fume)	29	(22.0)
Asbestos	11	(8.3)
Size of lymph nodes		
Short‐axis on CT, mm (median, range)	8.0 (4.0–9.9)	
Short‐axis on EBUS images, mm (median, range)	8.0 (4.9–16.0)	
Lymph node location		
Upper paratracheal (2R)	2	(1.3)
Lower paratracheal (4R, 4L)	50	(33.6)
Subcarinal (7)	79	(53.0)
Hilar (10R, 10L)	4	(2.7)
Interlobar (11s, 11i, 11L)	14	(9.4)
Puncture times (median, range)	2 (1–7)	
Final diagnosis		
Malignant	68	(45.6)
Adenocarcinoma	42	(28.2)
Squamous cell carcinoma	15	(10.1)
Small cell carcinoma	4	(2.7)
Pleomorphic carcinoma	1	(0.7)
Non‐pulmonary malignancies	5	(3.4)
Lymphoma	1	(0.7)
Benign	81	(54.4)
Nonspecific lymph node	65	(43.6)
Sarcoidosis	9	(6.0)
IgG4 related disease	3	(2.0)
Cryptococcosis	1	(0.7)
Organizing pneumonia	1	(0.7)
Pneumoconiosis	1	(0.7)
Wegener granulomatosis	1	(0.7)
SUVmax on PET‐CT		
≥2.5	89	(59.7)
<2.5	24	(16.1)
Not evaluated	36	(24.2)

Data are presented as number or median (range).

CT, computed tomography; EBUS‐TBNA, endobronchial ultrasound‐guided transbronchial needle aspiration; PET, positron emission tomography; SUVmax, maximum standardized uptake value.

### SAR analysis on EBUS elastography

**Figure 2:**
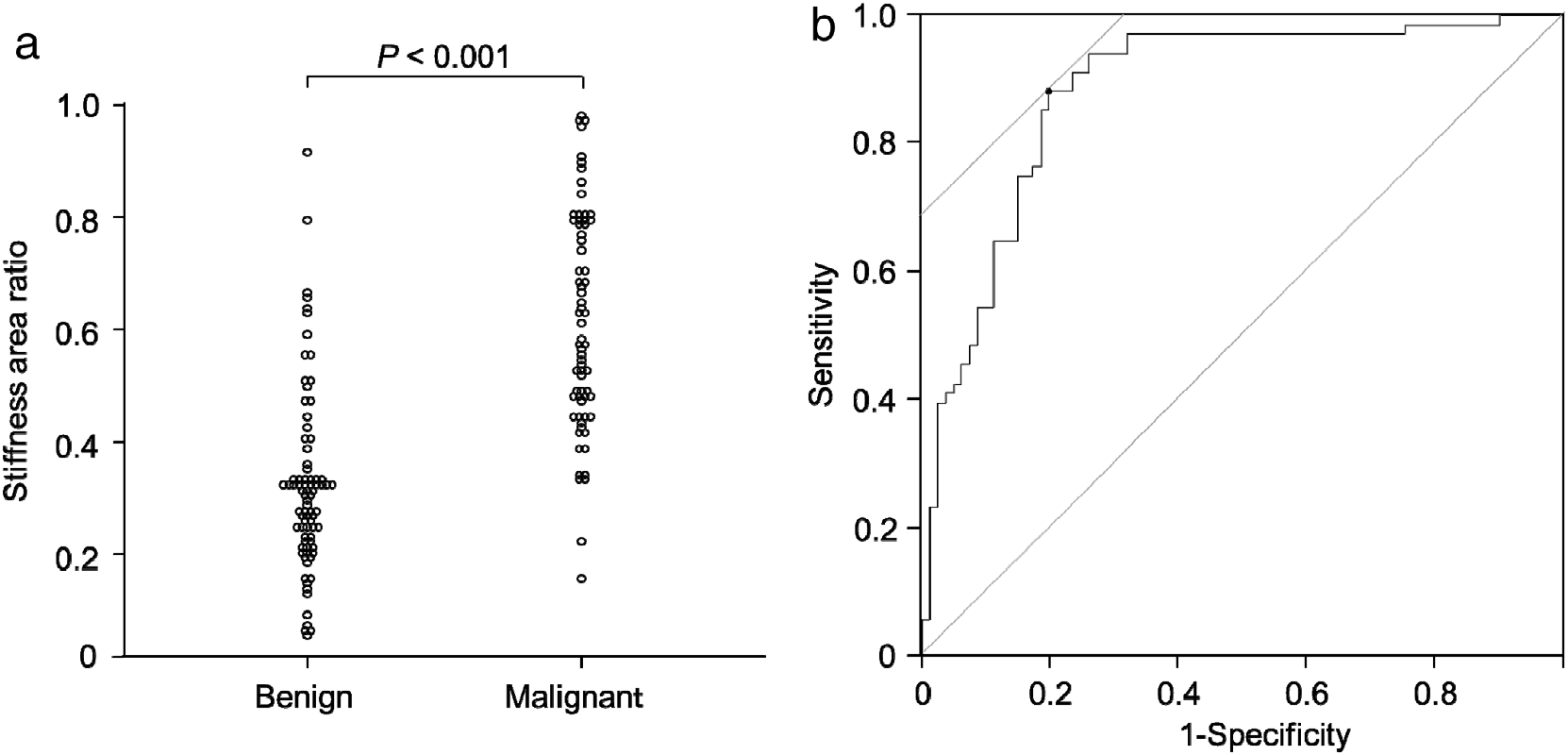
The stiffness area ratio of benign and malignant lymph nodes on EBUS elastography. (**a**) The median stiffness area ratio of malignant lymph nodes was higher than that of benign lymph nodes (0.58 vs. 0.31, *P* < 0.001, Mann‐Whitney U test). (**b**) A cutoff value of 0.41 was derived from the receiver operating characteristic curve for the stiffness area ratio. With this cutoff value, the sensitivity and specificity of elastography for predicting malignant lymph nodes were 88.2% and 80.2%, respectively. The area under the curve was 0.884 (95% confidence interval [CI]: 0.83–0.94).

Figure 2 shows the SARs in the LNs diagnosed as benign or malignant at the final diagnosis. The median SAR of malignant LNs was significantly higher than that of benign LNs (0.58 vs. 0.31, *P* < 0.001) (Fig 2a). The sensitivity and specificity of EBUS elastography for predicting malignant LNs were 88.2% (95% confidence interval [CI]: 78.1%–94.8%) and 80.2% (95% CI: 69.9%–88.3%), respectively, as assessed using a cutoff SAR value (0.41) calculated from the ROC curve. The AUC was 0.884 (95% CI: 0.83–0.94) (Fig 2b).

### B‐mode sonographic features and EBUS elastography

**Figure 3:**
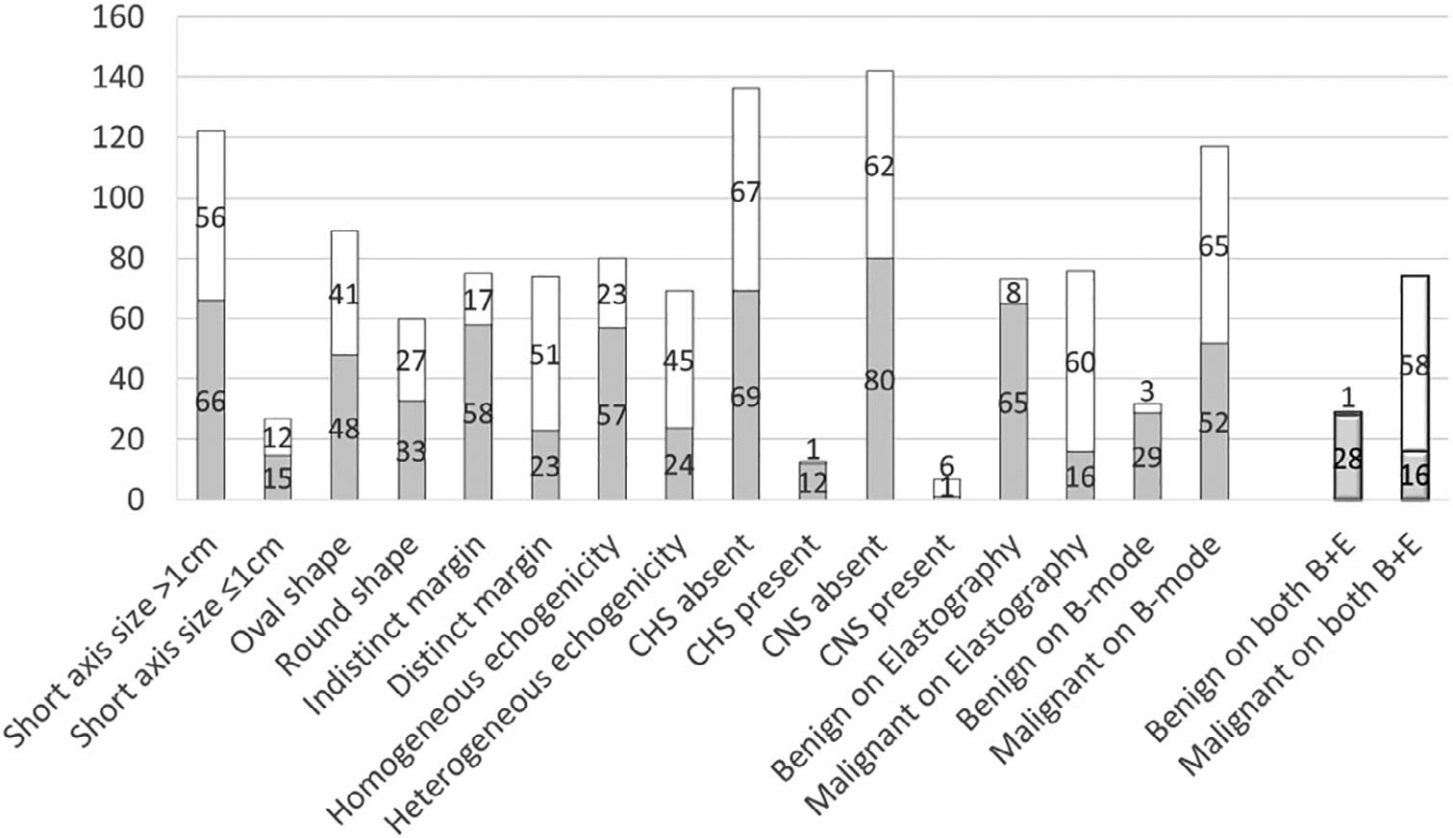
The distribution of benign and malignant lymph nodes based on the final diagnosis for each EBUS image category. B‐mode sonographic features were used to characterize LNs as benign, based on the absence of a (1) round shape, (2) heterogeneous echogenicity, (3) distinct margin, and (4) coagulation necrosis sign (CNS). On EBUS elastography, lymph nodes with a stiffness area ratio higher than 0.41 were judged as malignant. CHS refers to central hilar structure. B + E refers to combined B‐mode sonographic features and elastography (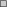) benign, (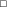) malignant.

Figure 3 demonstrates the number of LNs for each EBUS finding, and the benign/malignant status based on the final diagnosis. B‐mode sonographic and EBUS elastographic findings for all 149 LNs included the following: size (short‐axis) ≥1 cm in 27/149 cases (18.1%); round shape in 60/149 cases (40.3%); distinct margin in 74/149 (49.7%) cases; and heterogeneous echogenicity in 69/149 (46.3%) cases. EBUS elastographic and B‐mode sonographic features indicated malignancy in 76/149 (51.0%) cases and 117/149 (78.5%) cases, respectively. Combined B‐mode sonographic and EBUS elastographic features suggested malignancy in 74/149 (49.7%) cases, and benign lesions in 29/149 (19.5%) cases.

Table 2 summarizes the diagnostic test parameter for each EBUS finding in predicting malignant LNs. Univariate analysis revealed that malignant LNs could be significantly predicted based on the following factors: presence of a distinct margin (*P* < 0.001), heterogeneous echogenicity (*P* < 0.001), absence of CHS (*P* = 0.004), presence of CNS (*P* = 0.048), and malignancy on elastography (*P* < 0.001). Logistic regression analysis revealed that when EBUS elastography was suggestive of malignancy, the EBUS elastographic finding was the strongest independent predictor of malignant LNs (OR, 18.5; 95% CI: 6.48–52.6; *P* < 0.001). The sensitivity, specificity, PPV, NPV, and DAR were 88.2%, 80.2%, 78.9%, 89.0%, and 83.9%, respectively. Additionally, by adding B‐mode sonography to EBUS elastography, the sensitivity of LN malignancy increased to 98.3% (58/59) and the NPV increased to 96.6% (28/29).

**Table 2:** Diagnostic test parameters in each EBUS image category for predicting malignant lymph nodes

EBUS image category	Sensitivity (%)	Specificity (%)	PPV (%)	NPV (%)	Diagnostic accuracy (%)	Univariate *P*‐value^†^	Multivariate *P*‐value^‡^	Odds ratio(95% CI)^‡^
Size (>10 mm)	17.6	81.5	44.4	54.1	52.3	1.0	0.129	0.31 (0.07–1.40)
Shape (round)	39.7	59.3	45.0	53.9	50.3	1.0	0.004	4.77 (1.64–13.9)
Margin (distinct)	75.0	71.6	68.9	77.3	73.2	<0.001	0.045	3.16 (1.03–9.73)
Echogenicity (heterogeneous)	66.2	70.4	65.2	71.3	68.5	<0.001	0.506	1.45 (0.49–4.33)
CHS (absence)	98.5	14.8	49.3	92.3	53.0	0.004	0.054	10.1 (0.95–107)
CNS (presence)	8.8	98.8	85.7	56.3	57.7	0.048	0.417	3.03 (0.21–43.8)
Elastography (malignant)	88.2	80.2	78.9	89.0	83.9	<0.001	<0.001	18.5 (6.48–52.6)
B‐mode sonographic features (malignant)								
	95.6	35.8	55.6	90.6	63.1	<0.001		
Combination of elastography and B‐mode sonographic features (malignant)								
	98.3	63.6	78.4	96.6	83.5	<0.001		

Data are presented as percentages.

^†^ Calculated using Fisher's exact test. ^‡^Calculated using logistic regression analysis.

CHS, central hilar structure; CI, confidence interval; CNS, coagulation necrosis sign; EBUS, endobronchial ultrasound; NPV, negative predictive value; PPV, positive predictive value.

Table 3 summarizes the frequency of malignant LNs based on the short‐axis diameter on EBUS and CT. Larger LN size on EBUS was significantly associated with the frequency of malignancy (*P* = 0.046). On EBUS images, all three LNs with a short‐axis diameter less than or equal to 5.0 mm were finally diagnosed as benign. The median short‐axis size of the false negative LNs on EBUS elastography was 9.3 mm.

**Table 3:** Frequency of malignancy according to lymph node size on EBUS and CT

Size of lymph node	Number of malignant lymph nodes/total number (%)	*P*‐value^†^
Short‐axis diameter on EBUS (mm)		
≤5.0	0/3 (0)	0.046
5.1–6.0	3/11 (27.3)	
6.1–7.0	9/25 (36.0)	
7.1–8.0	23/41 (56.1)	
8.1–9.0	8/24 (33.3)	
≥9.1	25/45 (55.6)	
Total	68/149 (45.6)	
Short‐axis diameter on CT (mm)		
≤5.0	3/9 (33.3)	0.19
5.1–6.0	4/14 (28.6)	
6.1–7.0	13/27 (48.1)	
7.1–8.0	16/35 (45.7)	
8.1–9.0	16/32 (50.0)	
9.1–9.9	16/32 (50.0)	
Total	68/149 (45.6)	

Data are presented as number (percentage).

^†^ Calculated using the Cochran‐Armitage test for trend.

CT, computed tomography; EBUS‐TBNA, endobronchial ultrasound‐guided transbronchial needle aspiration.

## Discussion

This is the first study to compare detailed EBUS findings, including elastography, with pathological diagnoses in CT‐negative LNs. The results suggested that EBUS elastography can be more predictive of malignancy compared to other B‐mode findings. Combined evaluation using EBUS elastography and B‐mode sonography in CT‐negative LNs yielded the highest NPV for predicting malignancy in small‐sized mediastinal and hilar LNs.

Various EBUS elastography evaluation methods for mediastinal and hilar LNs have been described.^14^, ^15^, ^16^, ^17^, ^18^, ^19^, ^20^, ^21^, ^22^, ^23^ No differences were found in the diagnostic yields between quantitative and qualitative evaluations of EBUS elastography in a prospective study.^21^ In a comparison of quantitative evaluation (SAR or strain ratio) and qualitative evaluation (3‐image pattern classifications) of EBUS elastography, a meta‐analysis reported a significantly higher specificity for qualitative evaluation (90% vs. 81%, *P* = 0.001); however, the sensitivities were equivocal (92% vs. 93%, *P* = 0.74).^14^ While 3‐image pattern classifications are easy to evaluate and have been reported to be suitable for clinical practice,^15^, ^19^, ^20^ the definition and classification of intermediate (part blue, part non‐blue) LNs are ambiguous. As the intermediate classification includes LNs with widely varying SARs (and cutoff values), the values of the diagnostic test parameters with these 3‐image pattern classifications appear to be relatively high. Unlike qualitative tests, quantitative tests can eliminate ambiguous evaluations by using specific numerical values. To date, the only established quantitative evaluation method that can be performed in real time is the assessment of the strain ratio between the LNs and surrounding tissues.^17^ Nevertheless, the present study used a retrospective design, and the SAR was determined via existing medical records.

The present study demonstrated that quantitative evaluation of EBUS elastography using the SAR could accurately predict malignancy in CT‐negative LNs with a high NPV; this result is supported by two prior retrospective studies,^16^, ^22^ which used the same image processing software as the present study to evaluate the SAR. These two studies also reported similarly high NPVs (89.7%–90.3%) and DARs (78.5%–83.7%) for EBUS elastography.^16^, ^22^ However, the cutoff value of the SAR in the current study (41.2%) was higher than previously reported values (31.1%–36.7%)^16^, ^22^; this discrepancy might be attributed to the different inclusion rate of patients with specific medical and occupational histories, which are known to be associated with hardened LNs.

Small‐sized breast lesions have been reported to result in false negative diagnoses on breast ultrasonography with elastography.^28^ Conversely, in gastrointestinal endoscopic ultrasound elastography, small‐sized pancreatic tumors have been reported to result in fewer false positive diagnoses due to fewer surrounding inflammatory changes and ultrasound beam attenuation.^27^, ^29^ A prospective study of EBUS elastography using the strain ratio, reported that small intrathoracic LNs resulted in false negative diagnoses in patients with suspected lung cancer.^23^ In contrast, another prospective study of intrathoracic LNs conducted among 15 CT‐negative LNs reported that LN size did not affect the diagnostic test parameters of EBUS elastography.^17^ In the present study, the median short‐axis size of the false negative LNs on EBUS elastography was higher than the overall median size. Therefore, the false negative results on EBUS elastography may not be due to the smaller LN size.

LNs can be hardened due to calcification in patients with a history of pulmonary tuberculosis, pneumoconiosis, or anthracosis; this can result in false positive results for malignancy on EBUS elastography.^15^, ^17^, ^19^, ^20^ In this study, all six benign LNs with a SAR >0.6 were found in patients with a history of pulmonary tuberculosis or apparent exposure to mineral dusts such as coal or asbestos. The effectiveness of EBUS elastography has been reported in a population with a high prevalence of anthracosis^32^; however, the effects of other occupational inhalation exposures and a prior history of pulmonary tuberculosis on EBUS elastography remain poorly studied. It is important for bronchoscopists to acknowledge that a patient's occupational and medical history may affect the results of EBUS elastography; therefore, careful review and modification of the SAR cutoff value, based on each individual patient's history, are necessary. Although detailed occupational histories (e.g., inhalation exposure frequency, use of protective equipment) were unavailable in the present study, the findings of EBUS elastography were useful for differentiating benign and malignant CT‐negative LNs.

A retrospective study of 1061 mediastinal and hilar LNs reported that 96.0% (381/397) were benign, based on the following features: round shape, distinct margin, heterogeneous echogenicity, and CNS.^12^ However, the categorizations of each B‐mode finding have resulted in large differences in the diagnostic yields between studies,^12^, ^18^, ^19^, ^22^, ^23^ and the assessments have required appropriate training and experience.^16^ In contrast, EBUS elastography has demonstrated a high DAR in all studies,^14^, ^15^, ^16^, ^17^, ^18^, ^19^, ^20^, ^21^, ^22^, ^23^ and has been preferred by the operators due to the coloration of the tissues and their easy evaluation. Similar to the present study, recent reports have found improved diagnostic yields with the combination of elastography and B‐mode sonography.^18^, ^19^ Currently, the evaluation of LNs using the multiple EBUS image mode is indispensable for efficient EBUS‐TBNA.

Two recent meta‐analyses have reported the prevalence of occult LN metastasis to range from 12.8% to 15.0% in NSCLC patients with CT‐negative LNs.^10^, ^11^ In the present study, nearly half of small‐sized LNs (68/149, 45.6%) were finally diagnosed as malignant. This could be attributed to the inclusion of LNs with a high SUVmax. PET‐CT can assist the selection of target cases and lesions before EBUS‐TBNA, and improve the diagnosis of regional nodal involvement in NSCLC patients.^33^, ^34^ In this study, 4/24 (16.7%) of CT‐ and PET‐CT‐negative LNs were finally diagnosed as malignant, which was similar to previously reported rates (11.3%–18.4%).^10^, ^34^, ^35^

Small LNs on EBUS tended to be finally diagnosed as benign. To date, no studies have evaluated the final diagnosis (benign or malignant) of CT‐negative LNs based on 1 mm size increments on EBUS and CT. This is the first study to report that such differences in LN size affect the frequency of malignancy in CT‐negative LNs. Furthermore, the final diagnosis was benign in all LNs with a short‐axis diameter less than or equal to 5.0 mm on EBUS. This result is similar to that of a previous retrospective study, which reported a final benign diagnosis for all 14 patients with mediastinal LNs smaller than 5.0 mm on EBUS images.^36^ Despite the small sample size in this study and the use of a retrospective design, our results suggest that EBUS‐TBNA can be omitted in staging NSCLC patients when LNs are smaller than 5.0 mm on EBUS.

This study had several limitations. First, it utilized a single‐center retrospective design, and had a relatively small sample size. Second, the same bronchoscopist did not perform all the procedures; therefore, the skill of the bronchoscopist may have affected EBUS image analysis. Third, the BSIs and EEIs were only analyzed with static images; therefore, image selection bias may also have occurred. Fourth, this study also included LNs that had not been surgically removed in patients with lung cancer. Nevertheless, the true benignity of the LNs was clinically and radiologically evaluated. Further prospective, multicenter trials are required to confirm the results of this study.

In conclusion, the high DAR of EBUS elastography can predict malignancy in CT‐negative LNs with a higher NPV compared to B‐mode findings. Additionally, EBUS elastography demonstrated the highest NPV for malignancy, when used in combination with B‐mode sonography. Consequently, the combined evaluation of CT‐negative LNs using EEIs and BSIs may be helpful for bronchoscopists to perform EBUS‐TBNA efficiently.

## Disclosure

The authors declare that they have no competing interests.

## References

[1] Reck M , Rabe KF . Precision diagnosis and treatment for advanced non‐small‐cell lung cancer. N Engl J Med 2017; 377: 849–61. 28854088 10.1056/NEJMra1703413

[2] von Bartheld MB , Dekkers OM , Szlubowski A *et al* Endosonography vs conventional bronchoscopy for the diagnosis of sarcoidosis: The GRANULOMA randomized clinical trial. JAMA 2013; 309: 2457–64. 23780458 10.1001/jama.2013.5823

[3] Ye W , Zhang R , Xu X , Liu Y , Ying K . Diagnostic efficacy and safety of endobronchial ultrasound‐guided transbronchial needle aspiration in intrathoracic tuberculosis: A meta‐analysis. J Ultrasound Med 2015; 34: 1645–50. 26269299 10.7863/ultra.15.14.06017

[4] Silvestri GA , Gonzalez AV , Jantz MA *et al* Methods for staging non‐small cell lung cancer: Diagnosis and management of lung cancer, 3rd ed: American College of Chest Physicians evidence‐based clinical practice guidelines. Chest 2013; 143: e211S–50S. 23649440 10.1378/chest.12-2355

[5] Vilmann P , Clementsen PF , Colella S *et al* Combined endobronchial and esophageal endosonography for the diagnosis and staging of lung cancer: European Society of Gastrointestinal Endoscopy (ESGE) guideline, in cooperation with the European Respiratory Society (ERS) and the European Society of Thoracic Surgeons (ESTS). Endoscopy 2015; 47: 545–59. 26030890 10.1055/s-0034-1392040

[6] Wahidi MM , Herth F , Yasufuku K *et al* Technical aspects of endobronchial ultrasound‐guided transbronchial needle aspiration: CHEST guideline and expert panel report. Chest 2016; 149: 816–35. 26402427 10.1378/chest.15-1216

[7] Osarogiagbon RU , Lee YS , Faris NR , Ray MA , Ojeabulu PO , Smeltzer MP . Invasive mediastinal staging for resected non‐small cell lung cancer in a population‐based cohort. J Thorac Cardiovasc Surg 2019; 158: 1220–9 e2. 31147169 10.1016/j.jtcvs.2019.04.068 PMC6754300

[8] Gu P , Zhao YZ , Jiang LY , Zhang W , Xin Y , Han BH . Endobronchial ultrasound‐guided transbronchial needle aspiration for staging of lung cancer: A systematic review and meta‐analysis. Eur J Cancer 2009; 45: 1389–96. 19124238 10.1016/j.ejca.2008.11.043

[9] Adams K , Shah PL , Edmonds L , Lim E . Test performance of endobronchial ultrasound and transbronchial needle aspiration biopsy for mediastinal staging in patients with lung cancer: Systematic review and meta‐analysis. Thorax 2009; 64: 757–62. 19454408 10.1136/thx.2008.109868

[10] El‐Osta H , Jani P , Mansour A , Rascoe P , Jafri S . Endobronchial ultrasound for nodal staging of patients with non‐small‐cell lung cancer with radiologically normal mediastinum. A meta‐analysis. Ann Am Thorac Soc 2018; 15: 864–74. 29684288 10.1513/AnnalsATS.201711-863OC

[11] Leong TL , Loveland PM , Gorelik A , Irving L , Steinfort DP . Preoperative staging by EBUS in cN0/N1 lung cancer: Systematic review and meta‐analysis. J Bronchol Interv Pulmonol 2019; 26: 155–65. 10.1097/LBR.0000000000000545 30119069

[12] Fujiwara T , Yasufuku K , Nakajima T *et al* The utility of sonographic features during endobronchial ultrasound‐guided transbronchial needle aspiration for lymph node staging in patients with lung cancer: A standard endobronchial ultrasound image classification system. Chest 2010; 138: 641–7. 20382710 10.1378/chest.09-2006

[13] Nakajima T , Anayama T , Shingyoji M , Kimura H , Yoshino I , Yasufuku K . Vascular image patterns of lymph nodes for the prediction of metastatic disease during EBUS‐TBNA for mediastinal staging of lung cancer. J Thorac Oncol 2012; 7: 1009–14. 22525556 10.1097/JTO.0b013e31824cbafa

[14] Chen YF , Mao XW , Zhang YJ *et al* Endobronchial ultrasound elastography differentiates intrathoracic lymph nodes: A meta‐analysis. Ann Thorac Surg 2018; 106: 1251–7. 29738756 10.1016/j.athoracsur.2018.04.003

[15] Izumo T , Sasada S , Chavez C , Matsumoto Y , Tsuchida T . Endobronchial ultrasound elastography in the diagnosis of mediastinal and hilar lymph nodes. Jpn J Clin Oncol 2014; 144: 956–62. 10.1093/jjco/hyu105 25121724

[16] Nakajima T , Inage T , Sata Y *et al* Elastography for predicting and localizing nodal metastases during endobronchial ultrasound. Respiration 2015; 90: 499–506. 26571232 10.1159/000441798

[17] Korrungruang P , Boonsarngsuk V . Diagnostic value of endobronchial ultrasound elastography for the differentiation of benign and malignant intrathoracic lymph nodes. Respirology 2017; 22: 972–7. 28102963 10.1111/resp.12979

[18] Fujiwara T , Nakajima T , Inage T *et al* The combination of endobronchial elastography and sonographic findings during endobronchial ultrasound‐guided transbronchial needle aspiration for predicting nodal metastasis. Thorac Cancer 2019; 10: 2000–5. 31474004 10.1111/1759-7714.13186 PMC6775026

[19] Lin CK , Yu KL , Chang LY , Fan HJ , Wen YF , Ho CC . Differentiating malignant and benign lymph nodes using endobronchial ultrasound elastography. J Formos Med Assoc 2019; 118: 436–43. 30007831 10.1016/j.jfma.2018.06.021

[20] Huang H , Huang Z , Wang Q *et al* Effectiveness of the benign and malignant diagnosis of mediastinal and hilar lymph nodes by endobronchial ultrasound elastography. J Cancer 2017; 8: 1843–8. 28819382 10.7150/jca.19819 PMC5556648

[21] Sun J , Zheng X , Mao X *et al* Endobronchial ultrasound elastography for evaluation of intrathoracic lymph nodes: A pilot study. Respiration 2017; 93: 327–38. 28324873 10.1159/000464253

[22] Ma H , An Z , Xia P *et al* Semi‐quantitative analysis of EBUS elastography as a feasible approach in diagnosing mediastinal and hilar lymph nodes of lung cancer patients. Sci Rep 2018; 8: 3571. 29476168 10.1038/s41598-018-22006-4 PMC5824841

[23] Rozman A , Malovrh MM , Adamic K *et al* Endobronchial ultrasound elastography strain ratio for mediastinal lymph node diagnosis. Radiol Oncol 2015; 49: 334–40. 26834519 10.1515/raon-2015-0020 PMC4722923

[24] Moon WK , Chang RF , Chen CJ , Chen DR , Chen WL . Solid breast masses: Classification with computer‐aided analysis of continuous US images obtained with probe compression. Radiology 2005; 236: 458–64. 16040902 10.1148/radiol.2362041095

[25] Lyshchik A , Higashi T , Asato R *et al* Thyroid gland tumor diagnosis at US elastography. Radiology 2005; 237: 202–11. 16118150 10.1148/radiol.2363041248

[26] Castéra L , Vergniol J , Foucher J *et al* Prospective comparison of transient elastography, Fibrotest, APRI, and liver biopsy for the assessment of fibrosis in chronic hepatitis C. Gastroenterology 2005; 128: 343–50. 15685546 10.1053/j.gastro.2004.11.018

[27] Cui XW , Chang JM , Kan QC , Chiorean L , Ignee A , Dietrich CF . Endoscopic ultrasound elastography: Current status and future perspectives. World J Gatroenterol 2015; 21: 13212–24. 10.3748/wjg.v21.i47.13212 PMC4679753 26715804

[28] Choi HY , Seo M , Sohn YM *et al* Shear wave elastography for the diagnosis of small (≤2 cm) breast lesions: Added value and factors associated with false results. Br J Radiol 2019; 92: 20180341. 30817169 10.1259/bjr.20180341 PMC6580903

[29] Gonzalo‐Marin J , Vila JJ , Perez‐Miranda M . Role of endoscopic ultrasound in the diagnosis of pancreatic cancer. World J Gastrointest Oncol 2014; 6: 360–8. 25232461 10.4251/wjgo.v6.i9.360 PMC4163734

[30] Schneider CA , Rasband WS , Eliceiri KW . NIH image to ImageJ: 25 years of image analysis. Nat Methods 2012; 9: 671–5. 22930834 10.1038/nmeth.2089 PMC5554542

[31] Kanda Y . Investigation of the freely available easy‐to‐use software ‘EZR’ for medical statistics. Bone Marrow Transplant 2013; 48: 452–8. 23208313 10.1038/bmt.2012.244 PMC3590441

[32] Abedini A , Razavi F , Farahani M *et al* The utility of elastography during EBUS‐TBNA in a population with a high prevalence of anthracosis. Clin Respir J 2020; 14: 488–94. 10.1111/crj.13159. 32034995

[33] Yang DD , Mirvis E , Goldring J , Patel ARC , Wagner T . Improving diagnostic performance of 18F‐FDG‐PET/CT for assessment of regional nodal involvement in non‐small cell lung cancer. Clin Radiol 2019; 74: 818.e17–23. 10.1016/j.crad.2019.07.009 31420186

[34] Shin SH , Jeong BH , Jhun BW *et al* The utility of endosonography for mediastinal staging of non‐small cell lung cancer in patients with radiological N0 disease. Lung Cancer 2020; 139: 1515–6. 10.1016/j.lungcan.2019.11.021 31805443

[35] Serra P , Centeno C , Sanz‐Santos J *et al* Is it necessary to sample the contralateral nodal stations by EBUS‐TBNA in patients with lung cancer and clinical N0 / N1 on PET‐CT? Lung Cancer 2020; 142: 9–12. 32062200 10.1016/j.lungcan.2020.01.014

[36] Shingyoji M , Nakajima T , Yoshino M *et al* Endobronchial ultrasonography for positron emission tomography and computed tomography‐negative lymph node staging in non‐small cell lung cancer. Ann Thorac Surg 2014; 98: 1762–7. 25149044 10.1016/j.athoracsur.2014.05.078

